# The Dark Side of Orchid Symbiosis: Can *Tulasnella calospora* Decompose Host Tissues?

**DOI:** 10.3390/ijms21093139

**Published:** 2020-04-29

**Authors:** Martino Adamo, Matteo Chialva, Jacopo Calevo, Silvia De Rose, Mariangela Girlanda, Silvia Perotto, Raffaella Balestrini

**Affiliations:** 1 Department of Life Sciences and Systems Biology, University of Turin, Viale Mattioli 25, 10125 Turin, Italy; martino.adamo@unito.it (M.A.); matteo.chialva@unito.it (M.C.); silvia.derose@edu.unito.it (S.D.R.); mariangela.girlanda@unito.it (M.G.); silvia.perotto@unito.it (S.P.); 2 National Research Council, Institute for Sustainable Plant Protection, Viale Mattioli 25, 10125 Turin, Italy

**Keywords:** CAZymes, orchid mycorrhiza, gene expression, saprotrophic growth, orchid symbiosis

## Abstract

Photosynthetic orchids associate with mycorrhizal fungi that can be mostly ascribed to the “rhizoctonia” species complex. Rhizoctonias’ phylogenetic diversity covers a variety of ecological/nutritional strategies that include, beside the symbiosis establishment with host plants, endophytic and pathogenic associations with non-orchid plants or saprotrophic soil colonization. In addition, orchid mycorrhizal fungi (OMF) that establish a symbiotic relationship with an orchid host can later proliferate in browning and rotting orchid tissues. Environmental triggers and molecular mechanisms governing the switch leading to either a saprotrophic or a mycorrhizal behavior in OMF remain unclear. As the sequenced OMF genomes feature a wide range of genes putatively involved in the degradation of plant cell wall (PCW) components, we tested if these transitions may be correlated with a change in the expression of some PCW degrading enzymes. Regulation of several genes encoding PCW degrading enzymes was evaluated during saprotrophic growth of the OMF *Tulasnella calospora* on different substrates and under successful and unsuccessful mycorrhizal symbioses. Fungal gene expression *in planta* was investigated in two orchid species, the terrestrial Mediterranean *Serapias vomeracea* and the epiphytic tropical *Cattleya purpurata*. Although we only tested a subset of the CAZyme genes identified in the *T. calospora* genome, and we cannot exclude therefore a role for different CAZyme families or members inside a family, the results showed that the degradative potential of *T. calospora* is finely regulated during saprotrophic growth and in symbiosis, often with a different regulation in the two orchid species. These data pose novel questions about the role of fungal PCW degrading enzymes in the development of unsuccessful and successful interactions.

## 1. Introduction

Orchids associate with a diverse range of mycorrhizal fungi whose taxonomic position mainly correlates with the plant habitat and photosynthetic ability [1,2]. In particular, most photoautotrophic orchids form mycorrhizal associations with fungi in the ‘rhizoctonia’ species complex, a polyphyletic group that comprises teleomorphs in at least three families of Basidiomycetes: the Ceratobasidiaceae and Tulasnellaceae in the Cantharellales and the Serendipitaceae in the Sebacinales [3,4,5,6]. The Tulasnellaceae, in particular, are the most frequent orchid mycorrhizal fungi (OMF) found in both temperate and tropical regions [2]. There is a common belief that most orchid mycorrhizal (OM) rhizoctonias are unspecialized soil saprotrophs [7,8], based on observations of their fast growth in vitro on soluble sugars as well as complex polymers such as starch, pectin, cellulose and occasionally lignin [9,10,11]. However, recent studies reporting the absence/undetectability of some *Tulasnella* sp. outside the orchid rhizosphere [12,13] would question the actual saprotrophic capability of at least some OM rhizoctonias in the environment.

Although seed germination and early orchid development in Nature rely on mycorrhiza formation [14,15], the interaction of OM rhizoctonias with the orchid host shows a variety of possible outcomes. A compatible interaction leads to the formation of a mycorrhizal orchid protocorm, a postembryonic plant structure whose cells are colonized by intracellular fungal hyphae (*pelotons*). Viable mycorrhizal protocorms usually develop into photosynthetic seedlings, but they can occasionally be overgrown by the fungal mycelium and rot [9]. It has been suggested that unsuccessful mycorrhizal relationships may be due to the OM fungus switching from a biotrophic to a parasitic behavior [9]. A similar switch in growth behavior has been reported for *Serendipita vermifera* (syn. *Sebacina vermifera*), another OMF, during endophytic root colonization of *Arabidopsis* [16]. After a biotrophic phase of root colonization, *S. vermifera* is found to massively proliferate in dead or dying *Arabidopsis* root cells. Both interactions may occur simultaneously in a population of protocorms, thus emphasizing the dynamic and relatively unstable nature of the orchid-fungus association. However, the environmental and/or molecular clues leading to the different outcomes of plant-fungus interactions are currently unknown. It is also unclear whether the occasional rotting protocorms are the results of the OMF actually killing the orchid host as a necrotroph, or whether the fungus simply overgrows dead plant tissues as a saprotroph. In any case, both necrotrophic and saprotrophic growth would require an array of degradative enzymes in these fungi. Genome sequencing of fungi with different mycorrhizal abilities [17] has revealed a high number of Carbohydrate Active Enzymes (CAZymes) in the OMF *S. vermifera* and *Tulasnella calospora*. CAZymes are likely the most important enzymes involved in fungal and plant cell wall remodeling as well as in the degradation of plant-derived organic matter [18,19]. CAZymes have been grouped into four classes: glycoside-hydrolases (GH), glycosyl transferases (GT), polysaccharide lyases (PL) and carbohydrate esterases (CE), but several classes of auxiliary activities (AA) including redox enzymes acting together with CAZymes to degrade lignocellulose material were recently added to the ‘CAZy’ database (www.cazy.org [18]). In particular, the *T. calospora* (isolate AL13/4D) genome contains about one hundred CAZymes-coding genes, more than its saprotrophic sister taxon *Botryobasidium botryosum* [17]. Among these genes, several encode CAZymes and AAs involved in the degradation of plant cell wall (PCW) polysaccharides, including seven GH6 and twenty-seven GH7 (i.e., cellobiohydrolases with a role in the extensive saccharification of cellulose) in addition to thirty-three lytic polysaccharide monooxygenases (LPMOs) of the AA9 family [18]. It is unclear whether this wide array of CAZymes is mostly needed during saprotrophic growth of *T. calospora* because many CAZymes coding genes are also expressed by this fungus under symbiotic conditions [17]. A role of CAZymes during the development of the ectomycorrhizal (ECM) symbiosis has been suggested [19,20], and CAZymes may be needed to degrade the plant cell wall and to form intracellular fungal structures in endomycorrhizal associations [21].

The main aim of this study was to understand whether the expression of fungal CAZymes changed during saprotrophic growth of *T. calospora* on diverse substrates and in symbiosis with two different orchid species, the terrestrial *Serapias vomeracea* and the epiphytic *Cattleya purpurata*. In particular, available genomic and transcriptomic sequences of *T. calospora* isolate AL13/4D [17] allowed us to evaluate by RT-qPCR the gene expression of seven CAZyme coding genes belonging to GH and AA9 classes. Gradual browning and rotting of orchid protocorms colonized in vitro by *T. calospora* occurred occasionally for both host species, similarly to what has been reported for other orchid species [22,23,24]. Therefore, the expression of these fungal CAZymes was evaluated during saprotrophic growth as well as in the successful mycorrhizal interaction and in brown protocorms overgrown by the fungal mycelium. To monitor the plant-fungus interaction, the expression of fungal and plant marker genes expressed in symbiosis [25,26,27] was also tested. Unveiling the changes in *T. calospora* CAZyme gene expression under different conditions and on different host plants might provide new information on how this OMF switches from a symbiotic to a saprotrophic growth (e.g., in brown protocorms).

## 2. Results

### 2.1. Seed Germination and Microscopy Observations

**Figure 1:**
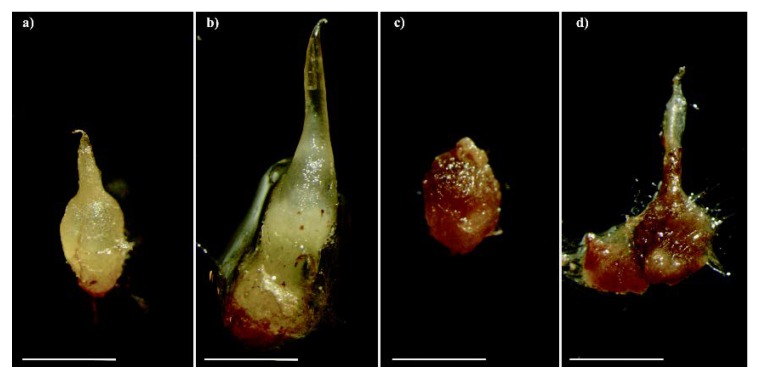
Protocorms of *Serapias vomeracea* inoculated with *Tulasnella calospora* at different stages: from the typical features (**a**) to a brown-dark/rot aspect (**b**–**d**). Bars = 2mm.

*Serapias vomeracea* seeds were germinated under both asymbiotic and symbiotic conditions. Asymbiotic germination on M551 medium yielded a germination percentage of 91.1% ± 6.9 SD, whereas symbiotic seed germination with *T. calospora* resulted in a germination percentage of 83.8% ± 5.3 SD. Occasionally, symbiotic *S. vomeracea* protocorms started to brown at stage P3 (development of a leaf projection) and eventually became very dark and soft (Figure 1).

**Figure 2:**
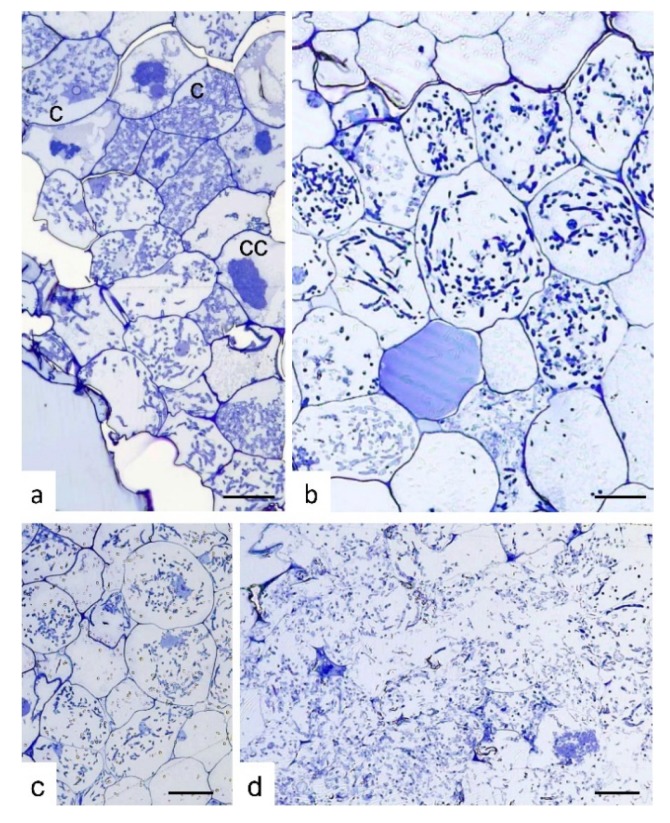
Semi-thin sections of *Serapias vomeracea* protocorms colonized by *Tulasnella calospora*. (**a**) Stage where protocorms appeared with the typical features and color. At cellular level, typical colonization pattern with *T. calospora* is evident with the presence of coils at different developmental stages. c, coil; cc, collapsed coil. (**b**,**c**) Subsequent stages where protocorms are becoming brown. The fungal colonization pattern is still evident as well as host cell features. (**d**) Section of a dark/soft protocorm. Cell borders are not well-defined and the fungal hyphae are widespread in the tissues without a typical colonization pattern. Bars = 33, 13, 45, 25 μm for (**a**), (**b**), (**c**) and (**d**), respectively.

Semi-thin sections of *S. vomeracea* protocorms colonized by *T. calospora* at different developmental and browning stages (selected on the basis of the external color, from white-yellowish to very dark brown) showed a typical intracellular mycorrhizal colonization only at early stages of browning (Figure 2a–c). By contrast, very dark and soft protocorms were colonized by fungal hyphae that were evenly distributed inside the protocorm tissues. In these protocorms, at least in some parts, plant cells were no longer surrounded by a well-defined cell wall (Figure 2d).

*Cattleya purpurata* seeds also germinated under both asymbiotic and symbiotic conditions. Asymbiotic germination on half-strength MS yielded a higher germination percentage (25.1% ± 3.2 SD) than symbiotic germination with *T. calospora* (18.3% ± 4.0 SD). Although the percentage of asymbiotically germinated seeds was higher for both *S. vomeracea* and for *C. purpurata*, the rate of orchid protocorms developing beyond the P3 stage was higher, in both orchid species, following symbiotic germination.

Healthy protocorms of *C. purpurata* developed into seedlings containing typical hyphal coils within root cells. Mycorrhizal root colonization was confirmed by staining with the FITC-Wheat Germ Agglutinin (WGA) conjugate, showing the presence of chitin in the fungal cell wall (Figure S1). Gradual browning and rot were occasionally observed also for *C. purpurata* protocorms colonized by *T. calospora*. These dark protocorms were overgrown by the fungal mycelium (Figure S1).

### 2.2. CAZymes Profiles in T. calospora and Other Basidiomycetes

**Figure 3:**
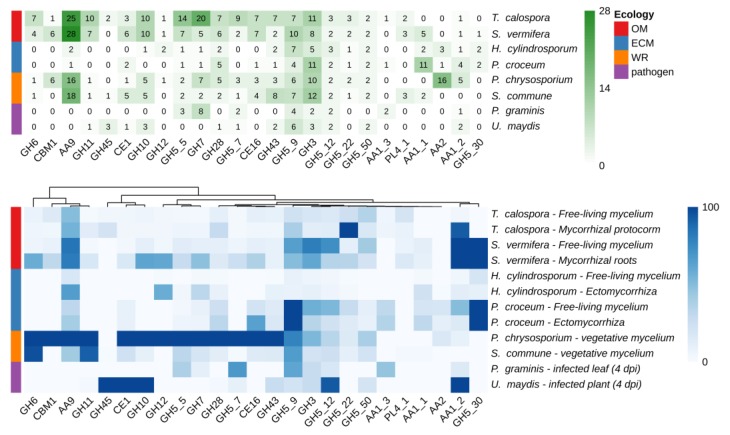
Number of CAZymes encoding-genes annotated in a number of Basidiomycota genomes (upper panel) and heatmap of expression levels across different tissues and conditions (bottom panel). Expression values are reported in TPM (transcripts per million) and columns are clustered by using Euclidean distances. Fungal species with different lifestyles were considered: orchid mycorrhizal (OM), ecto-mycorrhizal (ECM), white rot (WR), plant pathogens.

Taking advantage of available genomic and transcriptomic resources, the number of members inside the CAZyme gene families, as well as their expression levels, in *T. calospora* were compared with the data already available in different species of Basidiomycetes with different ecological and nutritional strategies, such as the OMF *S. vermifera*, two ECM fungi, two white rot and two phytopathogenic fungi [28,29,30,31,32,33,34]. Details on the species used, growing conditions and corresponding RNA-seq experiments accession are reported in Tables S1–S3. As shown in Figure 3, despite the highly populated CAZymes families annotated in the *T. calospora* genome, with some gene families being represented by very high gene numbers (e.g., GH5, GH6, GH7, GH10, GH11, AA9), there was in general a low global expression of these gene families both in symbiotic *S. vomeracea* protocorm tissues and in free-living mycelium grown on OA medium. By contrast, genes considered as being markers of saprotrophism and lignin degradation, such as GH5, GH6, GH7, GH11 and GH28, were highly expressed in the white rot fungus *P. chrysosporium* on a YMPG medium containing simple carbon sources [35].

### 2.3. CAZymes Expression in the Free-Living Mycelium and in Plant Tissues

The expression of ten *T. calospora* genes coding for GHs and AA9 CAZymes involved in the degradation of PCW polymers were tested by RT-qPCR on several growth conditions and developmental stages (Table 1).

A previous transcriptomic analysis [26] (Table 2) showed differential expression of these genes in free-living mycelium grown on OA medium and in mycorrhizal *S. vomeracea* protocorms. Here, we tested additional media and symbiotic conditions (Table 1). In detail, CAZymes expression during saprotrophic growth of *T. calospora* was evaluated on three different substrates: on MMN, a mineral medium supplemented with glucose as carbon source (a condition that should not induce CAZymes involved in the degradation of complex polysaccharides), on OA medium containing a mixture of starch and PCW polysaccharides, and on dried plant material (WS) that should mimic saprotrophic growth on dead host tissues (Table 1). Expression of these genes in association with the host plant was tested in *C. purpurata* and *S. vomeracea*, both in a compatible mycorrhizal interaction (SYM) and in protocorms exhibiting browning and rot (DP).

**Figure 4:**
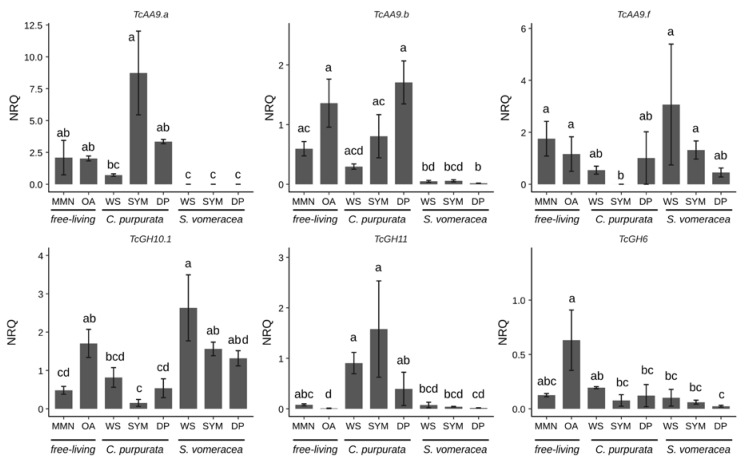
CAZymes expression under different experimental conditions. Letters indicate significant differences after Kruskal-Wallis test and Dunn’s post-hoc test (*p* < 0.05). NRQ, normalized relative quantities; mean ± standard error (SE) is plotted.

Among the AA9 genes tested in this work (Table S4), we focused on the three genes (TcAA9a, TcAA9b and TcAA9f) that resulted in detectable expression values by RT-qPCR (Figure 4).

Despite the high variability in the expression data, expression of all CAZymes was suppressed on the MMN medium (Figure 4). The three different genes coding for TcAA9 and *TcGH6* were expressed at the same levels both in MMN and OA samples, whereas *TcGH10* and *TcGH11* were significantly more/less expressed on OA than in MMN medium, respectively (Figure 4). A trend of decreasing expression, as compared with MMN and OA medium, is evident for *TcAA9a* and *TcAA9b* on dried plant material (WS), that was significant only for the *S. vomeracea* WS sample. By contrast, *TcAA9f* seemed more expressed in the *S. vomeracea* WS substrate, although this difference was not statistically significant (Figure 4). CAZyme expression during symbiotic growth of *T. calospora* was evaluated in both host plants. The results indicate, for most of the CAZyme genes, a different expression pattern in the two mycorrhizal plant species. This is particularly evident for the expression of *TcGH10*.1 and *TcGH11*, both involved in hemicellulose degradation, in the symbiotic (SYM) tissues of *C. purpurata* and *S. vomeracea* (Figure 4). In detail, the *TcGH11* gene was significantly downregulated in the S. vomeracea SYM samples, when compared to *C. purpurata*, whereas the opposite was observed for *TcGH10*.1 (Figure 4). Additionally, *TcGH11* was significantly upregulated, with respect to OA medium, in all growth conditions involving *C. purpurata* (both dead and living plant tissues). By contrast, *TcGH6* was not significantly regulated in any of the samples involving contact with plant tissues, independently from the plant species and the plant tissue being alive or dead (Figure 4). However, some differences could be observed when expression data were compared with saprotrophic growth on different media. For example, a lower *TcGH6* expression, with respect to the OA medium, was found in all the samples where *T. calospora* interacted with the plant, apart from the *C. purpurata* WS sample, whereas the differences were not significant when compared to MMN. When compared to *T. calospora* saprotrophic growth on different media as free living mycelium, TcGH10.1 expression showed an opposite trend with respect to *TcGH11*, with an expression level similar to OA in all the *S. vomeracea* samples (WS, SYM) and a downregulation in all the *C. purpurata* ones, with the lowest level in SYM sample. Expression of *TcAA9a* and *TcAA9b* was very low in symbiotic structures of *S. vomeracea*, independently form the status of the protocorms, while an upregulation trend is evident for the *C. purpurata* SYM samples. By contrast, *TcAA9f* was significantly downregulated in the *C. purpurata* SYM sample, suggesting a functional specificity. We also performed RT-qPCR with primers for *TcGH45*, which codes for a putative endoglucanase potentially active in cellulose degradation. However, a specific amplification signal could be observed only for *C. purpurata* samples, whereas the *S. vomeracea* samples yielded an aspecific signal. *TcGH45* expression was surprisingly higher on MMN (*p* ≤ 0.05), than on any other free-living conditions; the gene was upregulated in the SYM condition as compared to both OA and the WS samples (Figure S2).

**Figure 5:**
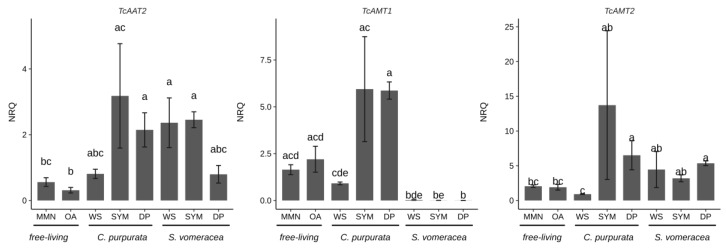
*T. calospora* symbiosis marker genes in *C. purpurata* and *S. vomeracea* orchid mycorrhiza interactions. Letters indicate significant differences after Kruskal-Wallis test and Dunn’s post-hoc test (*p* < 0.05). NRQ, normalized relative quantities; mean ± standard error (SE) is plotted.

To monitor the plant-fungus interaction in the plant tissues, we evaluated the expression of three fungal genes (*TcAAT2*, *TcAMT1*, *TcAMT2*) involved in the transport of nitrogen compounds and already demonstrated to be regulated in symbiosis [26,27]. In a similar way to the CAZymes, the expression level of the *TcAA2* and *TcAMT2* transporters was not significantly different in the plant samples (SYM and DP) colonized by *T. calospora*, for both plant species (Figure 5).

It is worth noting that *TcAMT1* showed an upregulation in *C. purpurata* tissues independently from their status (SYM or DP), while this trend was not found in *S. vomeracea*. Gene expression of the marker genes in the WS samples, i.e., free-living mycelium grown on dried plant material, was always similar to gene expression in the free-living mycelium grown on MMN and OA. For *S. vomeracea*, we also monitored the expression of two plant genes (*SvNOD1* and *SvEXO*) previously shown to be strongly upregulated in viable mycorrhizal protocorms [25]. The results (Figure S3) confirmed that both *SvEXO* and *SvNOD1* were upregulated in mycorrhizal (SYM) tissues with respect to asymbiotic protocorms (AP). Notably, the expression of these two plant genes was not significantly different in viable mycorrhizal (SYM) and in brown (DP) protocorm samples (Figure S2).

## 3. Discussion

In this study, we showed that symbiotic orchid seed germination with the OM fungus *T. calospora* led to contrasting outcomes in two orchid species, as already reported for other OM associations with fungi in the ‘rhizoctonia’ species complex [9,24]. *S. vomeracea* and *C. purpurata* seeds symbiotically germinated in vitro developed into healthy mycorrhizal protocorms featuring intracellular fungal pelotons, and subsequently developed (at least *C. purpurata*) into seedlings hosting typical fungal pelotons in the root cells. However, as already observed in other orchid systems [24], in both orchid species a variable percentage of mycorrhizal protocorms started to brown and rot in the plates and were eventually overgrown by the fungal mycelium. It has been suggested [9] that this unsuccessful mycorrhizal relationship may be due to the OM fungus switching from a biotrophic to a parasitic behavior. To assess whether the fungus had a role in the rotting protocorm phenotype, e.g., by increasing the production of PCW degrading enzymes, the transcriptional regulation of the fungal enzymatic abilities was verified, taking advantage of the information obtained in recent years on the mycorrhizal model system represented by *S. vomeracea* and *T. calospora* [25,26,27,36]. These previous papers were mainly focused on the symbiotic regulation of plant and fungal genes involved in N uptake and metabolism, but also led to the identification of genes specifically expressed in symbiotic protocorms (some of them only in *pelotons* containing cells) that can be considered symbiotic markers [25,26,27]. Transcriptomic analyses have been performed also on other orchid symbiotic systems (see [37] for a review), unveiling the orchid and fungal genes involved in symbiosis and providing novel useful information for a comprehensive understanding of OM biology. However, due to the complexity and the diversity of these interactions, several mechanisms involved in the developing and functioning of these associations remain to be elucidated.

In this study, in addition to the expression of different CAZyme families, we have investigated the expression of some fungal and plant genes previously identified as markers for the OM symbiosis. In particular, Fochi et al. [26] found differential expression in symbiosis of a repertoire of fungal genes involved in the transport of N compounds. These induced genes can therefore be considered as markers of the compatible interaction. Despite the broad data distribution, likely due to the biological systems considered and the difficulties to exactly synchronize the sample stages, both *TcAMT1* and *TcAMT2* appeared to be upregulated in mycorrhizal *C. purpurata* roots (SYM) as compared to the free-living mycelium grown on MMN, OA and WS. This finding, observed on different orchid hosts (*C. purpurata* and *S. vomeracea*) and at a different symbiotic stage (mycorrhizal roots for *C. purpurata* and mycorrhizal protocorms for *S. vomeracea*), confirms AMT genes as symbiosis markers for *T. calospora* [26]. An upregulation trend of *TcAMT2*, but not *TcAMT1*, in mycorrhizal *S. vomeracea* protocorms confirms previous results and suggests a slightly different relationship between the host plant and the fungus in terms of N transport.

Of the two plant genes previously shown to be upregulated in mycorrhizal *S. vomeracea* protocorm cells, *SvNOD1* and *SvEXO* [25,27], *SvNOD1* in particular was confirmed to be strongly upregulated in the mycorrhizal protocorms. The similar upregulation observed for both plant genes in brown protocorms, where the fungal hyphae proliferate inside the orchid tissues without forming typical coils, is intriguing and it may be due to the persistence of the corresponding gene transcripts in these plant tissues, or to the persistence of some intact cells in the rotting protocorms.

Despite the high variability, the picture emerging from the RT-qPCR experiments would exclude that the switch from a successful OM plant-fungus relationship to an unsuccessful one (i.e., the browning and rotting protocorms) correlates with a massive increase in the expression of fungal enzymes capable of degrading the host plant cell wall. In fact, quite unexpectedly, the expression of most CAZymes was not significantly different in viable mycorrhizal SYM samples (symbiotic protocorms for *S. vomeracea* and mycorrhizal roots for *C. purpurata*) and in brown, dark (DP) protocorms, for both plant hosts (Figure S4). Moreover, *TcGH45* gene expression in *C. purpurata* protocorms was significantly higher in viable mycorrhizal samples than in brown protocorms. As for the other CAZymes, some differences in transcript levels in DP with respect to SYM samples were observed, but values were not statistically different.

In the *T. calospora* CAZyme repertoire, cellulolytic activities are represented by enzymes of the GH5_5, GH12, GH45 (endoglucanases), cellobiohydrolases or exo-1,4-β-glucanases (GH6 and GH7) and AA9 (lytic polysaccharide mono-oxygenases that oxidatively cleave cellulose) families [17]. When interacting with *C. purpurata*, *T. calospora* upregulates *TcGH45* gene expression only when in symbiosis (SYM sample), suggesting for this CAZyme a role during mycorrhizal establishment or functioning, rather than during saprotrophic growth. This observation recalls other mycorrhizal systems, like the ECM formed by *Tuber melanosporum*, where the upregulation of a GH45 gene suggests a role in ECM development [20]. A recent study on the ECM fungus *Laccaria bicolor* suggests that a GH5 endoglucanase may have an important role in reshaping the plant cell wall during symbiosis [38], and it has been speculated that endoglucanases may be responsible for plant-cell remodeling during early fungal colonization on poplar [19].

The expression pattern of *TcGH6* suggests that this cellulase is probably not involved in the development of the symbiotic interaction but is used by *T. calospora* during saprotrophic growth. With respect to expression on the OA reference medium, *TcGH6* was significantly downregulated in the SYM samples in both species and may reflect the fungal ability to degrade plant-derived polysaccharides. However, a similar downregulation was also observed in the WS samples, containing dead orchid tissues, suggesting either that the fungus can recognize the host tissues thus exhibiting a “friendly” behavior, or that orchid tissues may contain compounds able to inhibit expression of some degradative enzymes in *T. calospora*. Orchid tissues can accumulate secondary metabolites that can affect fungal growth [39].

Lytic polysaccharide mono-oxygenases (LPMO) are CAZymes with auxiliary activities (AA9, formerly GH61) that often form a large multigene family in fungi and are active on the plant cell walls [40]. Fungal AA9 enzymes improve cellulase activity by the oxidation of crystalline cellulose and they randomly cleave cellulose chains at the microfibrils surface, thus loosening the cellulose microfibril structure [19]. An involvement of the different AA9 members in mycorrhizal formation was already demonstrated during ECM development [19]. Sillo et al. [20] showed that one of the two LPMO genes in the *Tuber melanosporum* genome was upregulated in ECM with respect to the free-living mycelium, and regulation of AA9 genes has been reported also at different stages of the interaction between *L. bicolor* and poplar [19]. Here, we showed the results for three different *T. calospora* AA9 genes: two of them, *TcAA9a* and *TcAA9b* yielded a significantly different expression in the two orchid species. In particular, a very low level of transcripts was found for both genes in all samples containing *S. vomeracea* tissues (WS, SYM, DP), irrespective of the tissues being alive or dead. These results would again suggest that *S. vomeracea* tissues may contain some compounds capable of specifically inhibit expression of these genes. By contrast, *TcAA9f* was expressed at the same level in the different conditions in *C. purpurata*, although a downregulation trend in DP with respect to SYM was observed in mycorrhiza *S. vomeracea* tissues.

Hemicelluloses are mainly represented by xylan, xyloglucan and galactomannan that cross-link cellulose microfibrils. In particular, xylan degradation is performed by a large set of enzymes including endo-β-1,4-xylanase/endo-β-1,3-xylanase belonging to GH10 and GH11 families. Here, two *T. calospora* genes (*TcGH10.1* and *TcGH11*) have been analyzed, showing only a weak regulation in the several conditions considered. It is worth noting that, as for the AA9 genes, a different regulation trend was evident in *C. purpurata* and *S. vomeracea* tissues. This observation could be due to a different symbiotic interaction of *T. calospora* with the two orchid species, but we cannot exclude that their expression mirrored a different cell wall composition for the two plant species.

## 4. Materials and Methods

### 4.1. Fungal Material and Growth as Free-living Mycelium

*Tulasnella calospora* AL13/D was isolated from the roots of the Mediterranean orchid *Anacamptis laxiflora* collected in Northern Italy as described in Girlanda et al. [4] but found to induce seed germination also on other plant species, like *Serapias vomeracea* [25,26] and *Cattleya purpurata* (this study). Three different free-living growth conditions were tested for *T. calospora*. The mycelium was grown on: (1) a modified Melin-Norkrans liquid medium (CaCl_2_ × 2H_2_O 0.066 g/L, NaCl 0.025 g/L, MgSO_4_ × 7H_2_O 0.15 g/L, FeCl_3_ × 6H_2_O 0.001 g/L, glucose 12.5 g/L, thiamine HCl 1.0 mL/L, l-glutamine 0.322 mL/L), as a control condition with a simple carbon source (hereinafter “MMN” experimental condition); (2) oatmeal agar medium [41], which contains a complex mixture of plant polysaccharides (“OA” condition); and (3) oven dried (at 70 °C for 24 h) asymbiotically grown orchid tissues (*C. purpurata* seedlings or *S. vomeracea* protocorms), to reproduce possible degradation of dead host tissues by *T. calospora*. In this last case, a 5 mm diameter mycelium plug was placed directly on the dead plant tissues, laid on oven-sterilized (180 °C, 3 h) quartz sand in 3 cm diam Petri dishes. Three mL of ddH_2_O was added to the quartz sand in the dishes to ensure sufficient moisture was present for the duration of the experiment.

### 4.2. Orchid Seed Germination and Symbiotic Fungal Growth

Capsules of *S. vomeracea* were collected in the Liguria Region, Italy (Pompeiana, IM; 43°51′16″92 N, 07°53′27″24 E). Collection was performed according to the Regional Law n. 28/2009. Capsules of *C. purpurata* were obtained by hand-pollinated plants belonging to the private collection of “Azienda Agricola Nardotto e Capello” (Camporosso, Imperia, Italy). At the time of capsule maturation, indicated by a yellowish color, seeds were collected into a paper sachet and then stored at 4 °C until use [42]. Symbiotic seed germination was obtained as described by Ercole at al. [40]. Briefly, after surface sterilization in 1% Sodium Hypochlorite solution for 20 min, orchid seeds were rinsed three times in sterile dH_2_O for 5 min and then sowed on oatmeal agar (OA) medium inoculated with a central 5 mm diameter. plug of the fungal isolate *T. calospora* AL13/4D previously maintained on solid 2% malt extract agar at 25 °C. *S. vomeracea* asymbiotic protocorms, as germination control, were obtained on Malmgren modified medium (M551, Phytotechnology), while *C. purpurata* asymbiotic protocorms, as germination control, were obtained on half-strength Murashige and Skoog [43] medium including vitamins (Duchefa, RV Haarlem, The Netherlands) and enriched with 50 mL/L of coconut water, 10 gr/L of sucrose and 2 gr/L activated charcoal. After germination, symbiotic protocorms were cultivated on OA medium. Whereas *C. purpurata* protocorms developed to the seedling stage and mycorrhizal roots were collected, *S. vomeracea* showed a slower development and mycorrhizal protocorms were collected at stage P3 [44]. Symbiotic tissues were indicated as SYM samples. Seed germination of both *S. vomeracea* and *C. purpurata* in combination with *T. calospora* resulted in occasional gradual browning of some protocorms (DP, dark protocorms). Different stages of browning *S. vomeracea* protocorms were collected and analyzed by microscopical observations.

### 4.3. Microscopy

Protocorms of *S. vomeracea* colonized by *T. calospora* at different developmental and browning stages (from white protocorms to very dark ones) were separately fixed in 2.5 glutaraldehyde in phosphate buffer 10 mM, washed in buffer and post-fixed in 1% osmium tetroxide. Samples were then dehydrated in an ethanol series (30%, 50%, 70%, 90%, 100% twice) and acetone 100% (twice) before embedding them in Epon/Araldite resin. Semi-thin sections were prepared and stained with Toluidine blue in order to verify the type of colonization by the fungus using a Carl Zeiss Primo Star optical microscope equipped with a DFC425C digital camera (Leica Microsystems GmbH, Wetzlar, Germany).

Root colonization of mycorrhizal seedlings of *C. purpurata* was verified by staining with FITC- wheat germ agglutinin (WGA) conjugate. Hand-cut root sections were incubated with 2% (*w/v*) BSA in PBS for 30 min at room temperature, washed three times with PBS/Tween 20 and incubated with FITC-conjugated WGA (0.1 mg/mL in PBS, 1% (*w/v*) (BSA) for 1 h at room temperature [45]. After three washes with PBS/Tween 20, colonized roots were mounted in distilled water and checked using an Eclipse E400 epifluorescence microscope (Nikon Instruments Inc., Amstelveen, The Netherlands).

### 4.4. RNA Extraction and cDNA Synthesis

Once the free-living *T. calospora* mycelium and the symbiotic plants were grown enough, they were collected and stored at −80 °C. RNA was extracted from each experimental condition following using a modified version of the “pine-tree” bench protocol by Chang et al. [46]. The extracted RNA was quantified using a ND-1000 Nanodrop (Thermo Fisher Scientific, Waltham, MA, USA) and treated with RNase-free DNase (TURBO DNA-free™ Kit Thermo Fisher Scientific), according to the manufacturer’s instructions. Approximately 500 ng of RNA were retrotranscribed in cDNA using SuperScript™ II Reverse Transcriptase (Thermo Fisher Scientific). cDNAs were diluted 1:2 and stored at −20 °C. For each experimental condition, three biological replicates were processed with the same procedure.

### 4.5. Primers Design and RT-qPCR

CAZymes specific primers, designed on the *T. calospora* genome sequences [17], were created using NCBI/Primer-BLAST based on Primer3 and BLAST (www.ncbi.nlm.nih.gov/tools/primer-blast/). Five representative families were considered (GH6, GH10, GH11 and GH45 and AA9), which are active on cellulose and hemicelluloses [18]. The genes were selected on the basis of their differential expression in symbiotic *versus* free-living mycelia in a previous *T. calospora* transcriptomic study [26]. Primer specificity for PCR was tested in silico on the *T. calospora* transcriptome using ‘primersearch’ tool in EMBOSS software suite [47]. *TcAMT1*, *TcAMT2* and *TcAAT2* primers were selected from the same study as markers for the symbiotic condition. Thanks to the available *S. vomeracea* assembled transcriptome [25], two genes previously described as being involved in symbiosis, *SvNOD1* [25] and *SvEXO* [26,27], were also considered. Further primer details are reported in Table S4.

RT-qPCR reactions were performed with at least two technical repetitions for each biological replicate as described in [48]. qPCR reaction volume was 15 μL composed of 2.25 μL ddH_2_O, 2.25 μL forward primer (3 μM); 2.25 μL reverse primer (3 μM), 7.5 μL of iQ SYBRTM Green^®^ Supermix (Bio Rad, Hercules, CA, USA) or Rotor-Gene SYBR^®^ Green PCR Kit (Qiagen, Hilden, Germany) and 0.75 μL cDNA. RT-qPCR reactions were performed using a Rotor-Gene Q (Qiagen) thermocycler using the following cycling conditions: 10 min at 95 °C, 40 cycles of 15 s at 95 °C and 1 min at 60 °C. To ensure reaction specificity a melting curve was recorded at the end of each run with a heating rate of 0.5 °C per 10 s and a continuous fluorescence measurement. The expression of *S. vomeracea* target transcripts was quantified after normalization to the two reference genes *SvUBI* and *SvEF1α* (ubiquitin and an elongation factor, respectively) while *T. calospora* elongation factor (was used to normalize the expression of fungal target transcripts. The same genes have previously been used in Fochi et al. [26,27].

### 4.6. Statistical Analysis

Relative gene expression was calculated as described in [49] using take-off and amplification efficiency values as calculated by the Rotor-Gene Q software using the ‘comparative quantitation’ mode. Normalized Relative Quantities (NRQ) were calculated based on gene specific amplification according to Pfaffl [50]. Data normality and homoscedasticity were checked on NRQ values using Shapiro-Wilk and Levene’s test respectively using ‘shapiro.test’ in package ‘stats‘ and ‘leveneTest’ function in package ‘car’ [51] at *p* < 0.05. Differences between means of different conditions were assessed using the Kruskal-Wallis test (*p* < 0.05) and if significant, Dunn’s posthoc test was used for multiple comparisons (*p* < 0.05) using the ‘dunnTest’ function in ‘FSA’ R package [52]. Barplots were generated using ggplot2 R package [53]. All analyses were performed in R programming environment [54].

### 4.7. RNA-seq Expression Profiles of CAZymes in T. calospora and Other Basidiomycetes Species

The expression profiles of CAZymes in *T. calospora* were compared with expression of the orthologous genes in different Basidiomycota species with different ecological strategies, using RNA-seq dataset available in the Short Read Archive (SRA, NCBI) from previous studies. *T. calospora*, *Serendipita vermifera* were selected as OM fungi, *Hebeloma cylindrosporum* and *Piloderma croceum* as ECM fungi, *Phanerochaete crysosporium* and *Schizophyllum commune* as white rots (WR), *Puccinia graminis* and *Ustilago maydis* as pathogens. Details on RNA-seq libraries used in this analysis, including accession numbers and full meta-data, are reported in Table S1.

Libraries were downloaded from the Short Read Archive (SRA) as. fastq files using the ‘fasterq-dump’ command in the NCBI SRA-Toolkit v2.9.6-1 software. Reads were filtered and trimmed using BBDuk software within BBTools suite from the Joint Genome Institute (https://jgi.doe.gov/data-and-tools/bbtools/) setting ‘ktrim = r k = 23 mink = 5 hdist = 1 qtrim = rl trimq = 10 maq = 15 minlen = 76 t = 10′. The bulit-in comprehensive Illumina adapters database (file ‘adapters.fa’) was used to filter residual adapters. Cleaned reads were pseudo-mapped on reference transcriptomes using ‘salmon’ v 0.11.3 [55] which accounts for sequencing and GC content biases using ‘--seqBias --gcBias --numBootstraps 1000 --posBias --validateMappings’ parameters. Reads number details are reported in Table S2. Publicly available reference transcriptomes were downloaded from MycoCosm project at Joint Genome Institute [56] as summarized in Table S3. Transcripts per million (TPM) were imported into R programming environment [54] using the ‘tximport’ R library [57]. CAZymes were predicted on reference transcriptomes using the ‘dbCAN’ pipeline implemented in the standalone ‘run_dbcan.py’ script [58] setting ‘inputType’ to ‘meta’ and leaving other parameters to default values. Transcripts were annotated as CAZymes only if assigned to the same family by at least two methods within DIAMOND, HotPep and HMMER. Gene expression heatmaps were generated averaging TPM values for transcripts IDs annotated within each family. Plots were generated using the ‘pheatmap’ package v1.0.12 in R [59] and scaled by rows. Only selected CAZymes families were reported according to results previously reported by Kohler et al. [17].

## 5. Conclusions

An intriguing question is whether the several genes coding for PCW degrading enzymes found in OMF are needed mainly for saprotrophic growth or whether they also play important functions in symbiosis. Although we miss a complete picture of CAZymes expression under the different conditions tested, our observations provide a first evidence that the degradative potential of *T. calospora* is finely regulated during saprotrophic growth and in symbiosis, with a different regulation often observed in the two orchid species. Apart from the cellulase *TcGH6,* all other CAZyme genes were differentially regulated in the mycorrhizal protocorms formed by the two orchid species and colonized by *T. calospora*, indicating that expression of the fungal CAZymes highly depends on the host plant. The fact that expression of these degradative enzymes did not significantly increase when the fungus colonized brown and rotting protocorms raises some questions on the actual cause behind these unsuccessful interactions and calls for further studies dedicated to understanding the role of fungal and plant determinants in this process. It could be interesting to verify the expression of transcription factors reported as activators of plant cell wall degrading enzymes in other fungi as well as to understand the role of the environmental conditions (e.g., temperature) and the host plant in the activation/repression of their expression.

## Figures and Tables

**Table 1:** Experimental conditions considered in RT-qPCR experiments.

Experimental Condition	ID	Description
Symbiotic roots/protocorms	SYM	Roots of *C. purpurata* seedlings or *S. vomeracea* protocorms colonized by *T. calospora*
Dark colonized protocorm/seedlings	DP	Protocorms/young seedlings of *C. purpurata* or protocorms of *S. vomeracea*, colonized by *T. calospora*, turned brown/black
Free-living fungus on dead plant tissues	WS	Dead leaves obtained from asymbiotic growth, then aseptically dried and inoculated with *T. calospora*
Free-living fungus on OA	OA	Free-living *T. calospora* grown on a complex Oat-meal Agar medium
Free-living fungus on MMN	MMN	Free-living *T. calospora* grown on mineral Melin-Norkins modified medium

**Table 2:** CAZyme and symbiosis marker genes considered in RT-qPCR experiments.

Gene Name	Putative Function	Transcript ID ^†^	MYC Normalized Reads ^‡^	FLMNormalized Reads ^‡^
*Tulasnella calospora*				
*TcGH6*	cellobiohydrolase	69053	2.71	12.98
*TcGH10a*	endo-1,4-β-xylanase	14789	3.37	24.59
*TcGH11*	endo-1,4-β-xylanase	80414	5.43	0.62
TcGH45*	endoglucanase	224031	26.58	6.49
*TcAA9a*	Lytic polysaccharide monooxygenase (LPMO)	75481	3.45	12.18
*TcAA9b*	LPMO	6298	102.01	61.82
*TcAA9f*	LPMO	4643	16.35	0.33
*TcAMT1*	ammonium transporter	241330	584.63	324.12
*TcAMT2*	ammonium transporter	183841	525.27	184.32
*TcAAT2*	amino acid transporter	81605	363.85	77.20
*Serapias vomeracea*				
*SvNod1*	early nodulin 55-2, putative	DN89686_c0_g1_i1	-	-
*SvEXO*	exocyst subunit exo70	DN73752_c2_g2_i1	42.61	7.38

* Oligonucleotides worked only in the *Cattleya*-*Tulasnella* model. **^†^** *T. calospora* transcripts IDs refers to the AL13/D transcriptome deposited in JGI portal (Kohler et al. 2015) while *S. vomeracea* IDs refers to the assembled transcriptome [26] available at NCBI (accession GSE87120). ^‡^ MYC reads and FLM reads refer to the *T. calospora* RNA-seq transcriptome analysis [17,26]. MYC reads and FLM reads columns show the mean number of reads observed in symbiotic and free-living conditions, respectively.

## Supplementary Materials

**Supplementary Materials** can be found at https://www.mdpi.com/1422-0067/21/9/3139/s1. Figure S1, *Cattleya purpurata* seedlings colonized by *Tulasnella calospora*. Figure S2, Expression pattern of *TcGH45* in *C. purpurata* model system and under free-living conditions. Figure S3, Symbiosis marker genes in *S. vomeracea*. Figure S4, *T. calospora* gene expression clustering in *C. purpurata*, *S. vomeracea* and free-living condition. Table S1, Species and experimental conditions of RNA-seq studies considered for gene expression meta-analysis. Table S2, Reads number and mapping details of selected RNA-seq libraries from short read archive (SRA). Table S3, Reference JGI fungal transcriptomes used for mapping. Table S4, Selected primers used in RT-qPCR experiments.

## Abbreviations

OM	Orchid Mycorrhiza
OMF	Orchid Mycorrhizal Fungi
PCW	Plant Cell Wall
CAZyme	Carbohydrate-active enzyme
GH	Glycoside-Hydrolase
GT	Glycosyl Transferase
PL	Polysaccharide Lyase
CE	Carbohydrate Esterase
AA	Auxiliary Activities
LPMO	Lytic Polysaccharide Monooxygenase
ECM	Ectomycorrhiza
WGA	Wheat Germ Agglutinin
