# Supplementary material for: The Dark Side of Orchid Symbiosis: Can Tulasnella calospora Decompose Host Tissues?
Source: Int J Mol Sci. 2020 Apr 29;21(9):3139. doi: 10.3390/ijms21093139 (PMC7247694; doi:10.3390/ijms21093139)
Supplement: Supplementary file 1 [file ijms-21-03139-s001.pdf]

Supplementary Materials:

The Dark Side of Orchid Symbiosis: Can *Tulasnella calospora* Decompose Host Tissues?

Martino Adamo [1], Matteo Chialva [1], Jacopo Calevo [1,‡,*], Silvia De Rose [1], Mariangela Girlanda [1], Silvia Perotto [1,†] and Raffaella Balestrini [2,†,*]

[1] Department of Life Sciences and Systems Biology, University of Turin, Viale Mattioli 25, 10125 Turin, Italy; martino.adamo@unito.it (M.A.), matteo.chialva@unito.it (M.C.), silvia.derose@edu.unito.it (S.D.R.), mariangela.girlanda@unito.it (M.G.), silvia.perotto@unito.it (S.P.)
[2] National Research Council, Institute for Sustainable Plant Protection, Viale Mattioli 25, 10125 Turin, Italy
* Correspondence: raffaella.balestrini@ipsp.cnr.it (R.B.); jacopo.calevo@gmail.com (J.C.); Tel.: +390116502927 (R.B.)
† These authors contributed equally to this work.
‡ Present address: Department of Biology, University of Naples Federico II, campus Monte Sant'Angelo building 7, Via Cinthia, 80126 Naples, Italy

Supplementary Figures

Figure S1. *Cattleya purpurata* seedlings colonized by *Tulasnella calospora*.

Figure S2. Expression pattern of *TcGH45* in *C. purpurata* model system and under free-living conditions.

Figure S3. Symbiosis marker genes in *S. vomeracea*.

Figure S4. *T. calospora* gene expression clustering in *C. purpurata*, *S. vomeracea* and free-living condition.

Supplementary Tables

Table S1. Species and experimental conditions of RNA-seq studies considered for gene expression meta-analysis.

Table S2. Reads number and mapping details of selected RNA-seq libraries from short read archive (SRA).

Table S3. Reference JGI fungal transcriptomes used for mapping.

Table S4. Selected primers used in RT-qPCR experiments.

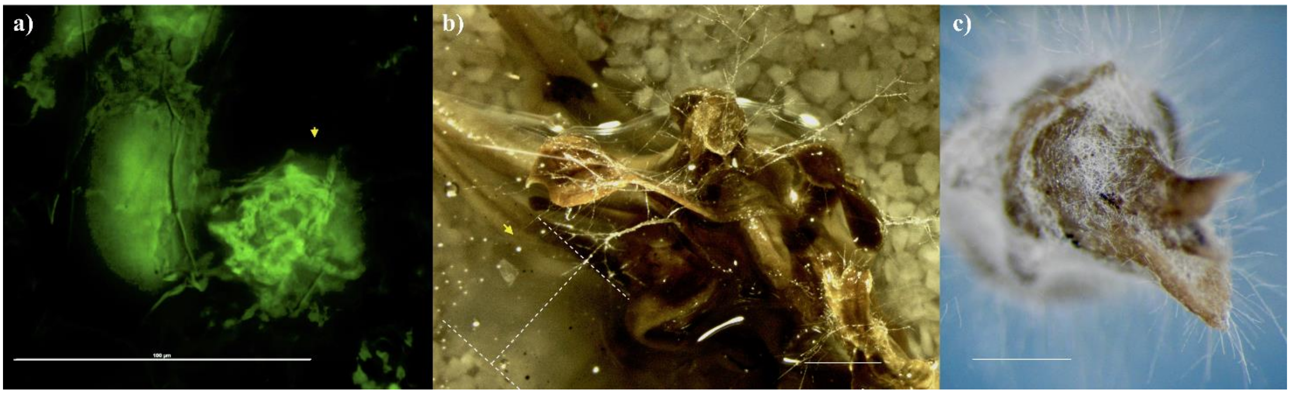

Figure S1. *Cattleya purpurata* seedlings colonized by *Tulasnella calospora*. **a**) Detail of a peloton (yellow arrow) inside the orchid root cells. Scale bar = 100 µm; **b**) dead orchid tissues covered by fungal hyphae. Fungal inoculum is indicated by a yellow arrow. Scale bar = 2 mm; **c**) young dead seedling where the saprotrophic behavior of the fungus seems to prevail. Scale bar = 3 mm.

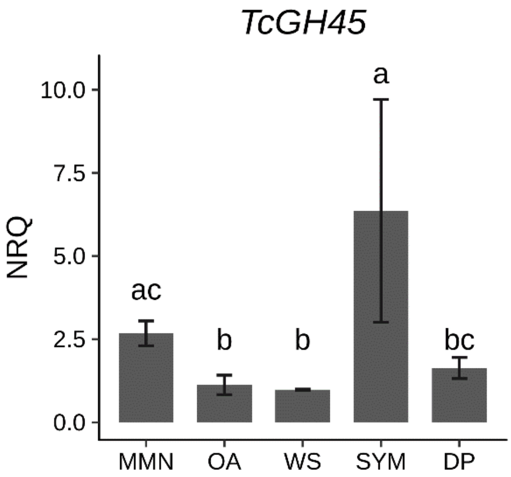

Figure S2. Expression pattern of *TcGH45* in *C. purpurata* model system and under free-living conditions. Letters indicate significant differences after Kruskal-Wallis test and Dunn's post-hoc test ($p < 0.05$). NRQ, normalized relative quantities; mean ± standard error (SE) is plotted.

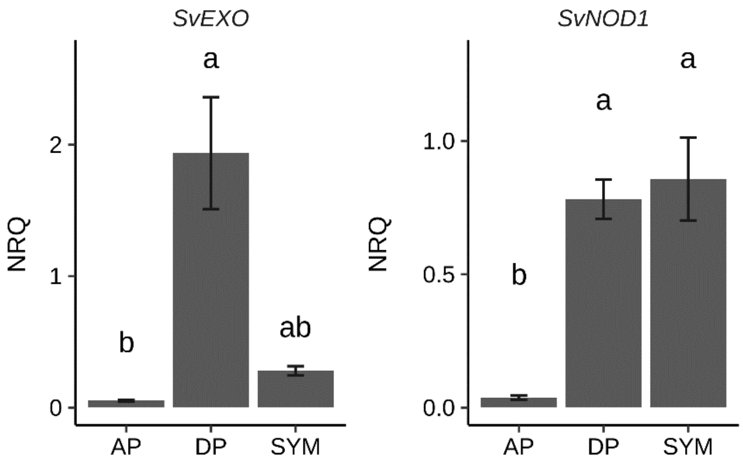

Figure S3. Symbiosis marker genes in *S. vomeracea*. Letters indicate significant differences after Kruskal-Wallis test and Dunn's post-hoc test ($p < 0.05$). NRQ, normalized relative quantities; mean ± standard error (SE) is plotted.

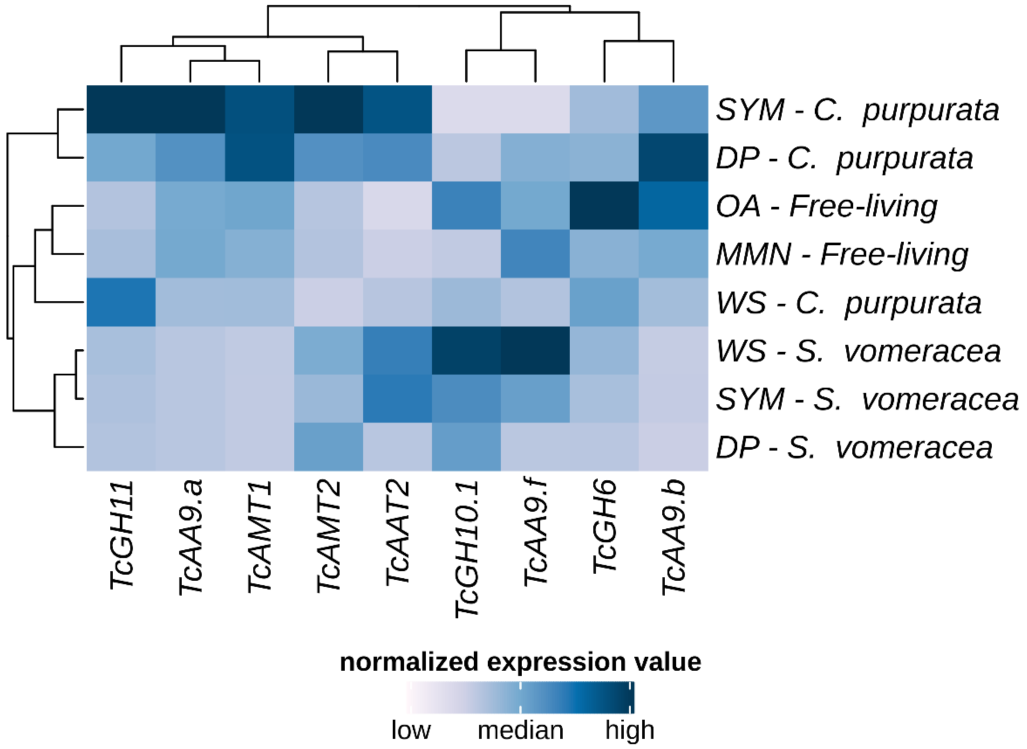

Figure S4. *T. calospora* gene expression clustering in *C. purpurata, S. vomeracea* and *free-living* condition. Heatmaps show z-score scaled NRQ values by gene from RT-qPCR experiments. Hierarchical clustering dendrograms show Pearson correlation of expression values across conditions and genes.

Table S1. Species and experimental conditions of RNA-seq studies considered for gene expression meta-analysis. Fungal species with different ecology were considered: orchid mycorrhizal (OM), ecto-mycorrhizal (ECM), white rot (WR), pathogens.

Species	Strain	Ecology	Conditions	Plant Host	Instrument and Layout	SRA Study	References
Tulasnella calospora	AL13	OM	free-living mycelium, protocorm	*Serapias vomeracea*	HiSeq2000, PE	SRP050561	Kohler et al. (2015)
Serendipita vermifera	MAFF 30583	OM	free-living mycelium, roots	*Arabidposis thaliana*	HiSeq2000, PE	SRP050866	Kohler et al. (2015)5
Hebeloma cylindrosporum	TV98 IV3	ECM	free-living mycelium, roots	*Pinus pinaster*	HiSeq2000, SE	SRP050559	Kohler et al. (2015)
Piloderma croceum	F 1598	ECM	free-living mycelium, roots	*Quercus robur*	HiSeq2000, PE	SRP050868	Kohler et al. (2015)
Phanerochaete chrysosporium	RP78	WR	vegetative mycelium	-	HiSeq2500, PE	SRP179770	Krizsán et al. (2019)
Schizophyllum commune	H4-8	WR	vegetative mycelium	-	HiSeq2500, PE	SRP179769	Krizsán et al. (2019)
Puccinia graminis f.sp. tritici	99KS76A-1	Pathogen	infected leaf (4 dpi*)	*Triticum aestivum*	Illumina Genome Analyzer, PE	SRP121504	Salcedo et al. (2017)
Ustilago maydis	FB1 x FB2	Pathogen	infected leaf (4 dpi*)	*Zea mays*	HiSeq2000, PE	SRP117697	Lanver et al. (2018)

* days post infection.

Table S2. Reads number and mapping details of selected RNA-seq libraries from short read archive (SRA).

SRA Run	Species	Average Read Length	Condition	Raw	Filtered	Mapped	Mapped (%)
SRR1693601	*Tulasnella calospora*	200	Free-living mycelium	16,495,698	14,788,396	7,537,645	50.97
SRR1693602	*Tulasnella calospora*	200	Free-living mycelium	29,808,337	26,571,893	19,400,139	73.01
SRR1693603	*Tulasnella calospora*	200	Free-living mycelium	23,835,798	21,445,992	16,663,536	77.7
SRR1693604	*Tulasnella calospora*	200	Mycorrhizal protocorm	24,757,364	22,887,687	2,085,068	9.11
SRR1693605	*Tulasnella calospora*	200	Mycorrhizal protocorm	26,783,635	24,835,443	2,200,420	8.86
SRR1693606	*Tulasnella calospora*	200	Mycorrhizal protocorm	28,131,331	26,299,612	2,422,194	9.21
SRR1695524	*Serendipita vermifera*	202	Free-living mycelium	40,981,314	34,350,150	26,645,411	77.57
SRR1695525	*Serendipita vermifera*	202	Free-living mycelium	42,985,842	35,737,581	28,297,017	79.18
SRR1695526	*Serendipita vermifera*	202	Free-living mycelium	15,118,764	12,620,290	10,050,799	79.64

SRR1695527	*Serendipita vermifera*	202	Mycorrhizal roots	6,047,711	4,973,626	72,118	1.45
SRR1695528	*Serendipita vermifera*	202	Mycorrhizal roots	7,767,250	6,429,432	76,510	1.19
SRR1695529	*Serendipita vermifera*	202	Mycorrhizal roots	6,304,164	5,200,967	46,809	0.9
SRR1693517	*Hebeloma cylindrosporum*	150	Free-living mycelium	36,570,502	36,097,183	26,751,622	74.11
SRR1693518	*Hebeloma cylindrosporum*	150	Free-living mycelium	35,884,216	35,583,533	24,826,631	69.77
SRR1693519	*Hebeloma cylindrosporum*	150	Free-living mycelium	33,590,534	33,229,737	23,024,885	69.29
SRR1693520	*Hebeloma cylindrosporum*	150	Ectomycorrhiza	104,061,378	101,762,901	36,807,641	36.17
SRR1693521	*Hebeloma cylindrosporum*	150	Ectomycorrhiza	90,962,146	89,468,417	27,529,432	30.77
SRR1693522	*Hebeloma cylindrosporum*	150	Ectomycorrhiza	99,771,880	98,001,116	32,085,565	32.74
SRR1695538	*Piloderma croceum*	202	Free-living mycelium	59,973,882	55,067,234	45,815,939	83.2
SRR1695539	*Piloderma croceum*	202	Free-living mycelium	16,176,073	12,951,125	10,821,960	83.56
SRR1695540	*Piloderma croceum*	202	Free-living mycelium	24,212,031	19,310,902	16,157,432	83.67
SRR1695541	*Piloderma croceum*	202	Ectomycorrhiza	22,510,865	19,224,485	2,868,293	14.92
SRR1695542	*Piloderma croceum*	202	Ectomycorrhiza	21,303,775	18,468,413	2,478,461	13.42
SRR1695543	*Piloderma croceum*	202	Ectomycorrhiza	21,366,078	18,418,760	2,562,050	13.91
SRR8448307	*Phanerochaete chrysosporium*	249	vegetative mycelium	23,244,895	23,141,396	17,064,465	73.74
SRR8448308	*Phanerochaete chrysosporium*	249	vegetative mycelium	48,638,574	48,414,251	36,020,203	74.4
SRR8448309	*Phanerochaete chrysosporium*	249	vegetative mycelium	27,240,214	27,127,794	20,299,728	74.83
SRR8448292	*Schizophyllum commune*	249	vegetative mycelium	23,834,135	23,668,839	18,594,240	78.56
SRR8448293	*Schizophyllum commune*	249	vegetative mycelium	26,519,052	26,318,179	20,915,057	79.47
SRR8448294	*Schizophyllum commune*	249	vegetative mycelium	25,878,484	25,711,912	20,096,430	78.16
SRR6218744	*Puccinia graminis*	202	infected leaf (4 dpi)	39,258,693	35,329,192	936,224	2.65
SRR6218745	*Puccinia graminis*	202	infected leaf (4 dpi)	50,449,094	42,319,457	922,564	2.18
SRR6218772	*Puccinia graminis*	202	infected leaf (4 dpi)	25,880,263	23,943,494	474,081	1.98
SRR6039731	*Ustilago maydis*	202	infected plant (4 dpi)	30,838,063	29,406,524	349,938	1.19
SRR6039732	*Ustilago maydis*	202	infected plant (4 dpi)	38.837.342	37.108.931	890.614	2.4
SRR6039733	*Ustilago maydis*	202	infected plant (4 dpi)	35.511.412	32.985.380	1.576.701	4.78

Table S3. Reference JGI fungal transcriptomes used for mapping.

Species	Version	Reference
Tulasnella calospora	Tulca1 AL13/4D v1.0	Kohler et al. (2015)
Serendipita vermifera	Sebve1 MAFF 305830 v1.0	Kohler et al. (2015)
Hebeloma cylindrosporum	Hebcy2 h7 v2.0	Kohler et al. (2015)
Piloderma croceum	Pilcr1 F 1598 v1.0	Kohler et al. (2015)
Phanerochaete chrysosporium	Phcr2 RP-78 v2.2	Ohm et al. (2014)
Schizophyllum commune	Schco3 H4-8 v3.0	Ohm et al. (2010)
Puccinia graminis f.sp. tritici	Pucgr2 f. sp. tritici v2.0	Duplessis et al. (2011); Cuomo et al. (2017)
Ustilago maydis	Ustma2 521 v2.0	Kamper et al. (2006)

Table S4. Selected primers used in RT-qPCR experiments. Gene description corresponds to the main activity of the gene. In the reference column, the original studies for primers sequences are reported. Reference genes primer names are reported in bold.

**Primer Name**	**Target Species**	**Description**	**Sequence (5′–3′)**	**Transcript ID†**	**Reference**
TcGH6.03	*Tulasnella calospora*	CAZyme - cellobiohydrolase	F - GCCACCCAGAGCAGTTCTAC<br>R - GCAAGGTAATCGCCGAGAGT	69053	This study
TcGH10a	*Tulasnella calospora*	CAZyme - endo-1,4-β-xylanase/endo-1,3-β-xylanase	F - CGGAGCCTTCGCAGAACTCG<br>R - GCTCGCGCTCGTGATAGACG	14789	This study
*TcGH11**	*Tulasnella calospora*	CAZyme - endo-1,4-β-xylanase/endo-1,3-β-xylanase	F - CGCTACTGCTGCCGTCTTGG<br>R - GCCGTCCGTCCAGAAGGAGT	80414	This study
TcGH11new	*Tulasnella calospora*	CAZyme - endo-1,4-β-xylanase/endo-1,3-β-xylanase	F - CTACAAGGCCATCCGAACCA<br>R - GTTCCGCCAGTACGATGACT	80414	This study
*TcGH45**	*Tulasnella calospora*	CAZyme - endoglucanase	F - CAGCAACCTTCCTGCGGCTC<br>R - AGAAGCGGGGCAAGTGACCT	224031	This study
TcAA9a	*Tulasnella calospora*	CAZyme - Lytic Polysaccharide Monooxygenase (LPMO)	F - CGTTAGCATCTTCTGGGGCA<br>R - CGATCTTGGTCGGGTCAGTC	75481	This study
TcAA9b	*Tulasnella calospora*	CAZyme - Lytic Polysaccharide Monooxygenases (LPMO)	F - ATCAGGCGAACCTTCTGTCC<br>R - ACTTGGCGTCGAGAGAGTTG	6298	This study
TcAA9c§	*Tulasnella calospora*	CAZyme - Lytic Polysaccharide Monooxygenases (LPMO)	F - CGGTCTGGTTCAAAGTTGCC<br>R - CGTGCAGAGCAATGTGTTCG	115568	This study
TcAA9d§	*Tulasnella calospora*	CAZyme - Lytic Polysaccharide Monooxygenases (LPMO)	F - CTCTGATCGTCAACGGCACT<br>R - AAGAAACGACTGTGACGGGG	27439	This study
TcAA9e§	*Tulasnella calospora*	CAZyme - Lytic Polysaccharide Monooxygenases (LPMO)	F - CAAGCCAACAATCCGCAACT<br>R - CTGATTGACTGGGGGTTGGG	641723	This study
TcAA9f	*Tulasnella calospora*	CAZyme - Lytic Polysaccharide Monooxygenases (LPMO)	F - GGAGACTGAAGGGATTGGGC<br>R - CGTCAGCCATGTTGCGTATG	4643	This study
TcAMT1	*Tulasnella calospora*	Ammonium transporter	F - AGGACCAACTACCGCCACCA<br>R - CATCCGTAAGGAGCGCCATC	241330	Fochi et al. (2017a)
TcAMT2	*Tulasnella calospora*	Ammonium transporter	F - ATTCGCGAGCTGCTTCCATC<br>R - GCAAGGTGGGGCTGAACATC	183841	Fochi et al. (2017a)

TcAAT2	*Tulasnella calospora*	Aminoacid transporter	F - AAAGGCGGTATTGCGCTTCA<br>R - GGCAGAGAAAGCACCGAGGA	81605	Fochi et al. (2017a)
TcEF1α	*Tulasnella calospora*	Elongation factor (fungal reference gene)	F - CAAGCCTATGTGCGTTGAGA<br>R - ACGAGAATGCGAAGGAAGAA	241957	Perotto et al. (2014)
SvNod1	*Serapias vomeracea*	Early nodulin 55-2, putative	F - TGACGGCATCCGCCGAGTTCT<br>R - ACTTGGAATCTCATACGCTCCGCCC	DN89686_c0_g1_i1	Perotto et al. (2014)
SvEXO	*Serapias vomeracea*	Exocyst subunit exo70 family protein H2	F - CTGCTTCGCCGATGTTACCC<br>R - TCGGAGAGGGCTTGGTGAAG	DN73752_c2_g2_i1	Fochi et al. (2017b)
SvUBI	*Serapias vomeracea*	Ubiquitin	F - TCTATCTATTCTTGCGGGGG<br>R - CACATGGCGCAGTCAATAAA	DN70641_c1_g2_i1	Perotto et al. (2014)
SvEF1α	*Serapias vomeracea*	Elongation factor (plant reference gene)	F - CGATCTCGTAGTGATCTGAGCAAG<br>R - GAGACAGAAATAACAACCAGCAACA	DN66666_c0_g1_i1	Fochi et al. (2017a)

Notes: * oligonucleotides that worked only in the *Cattleya-Tulasnella* model. § oligonucleotides tested, but not included in results due to low or no amplification. † *T. calospora* transcripts IDs refers to the AL13/D transcriptome deposited in JGI portal (Kohler et al. 2015) while *S. vomeracea* IDs refers to the assembled transcriptome (Fochi et al. 2017a) available at NCBI (accession GSE87120). ‡ MYC reads and FLM reads refer to the *T. calospora* RNA-seq transcriptome analysis (Kohler et al. 2015; Fochi et al. 2017a). MYC reads and FLM reads columns show the mean number of reads observed in symbiotic *T. calospora* and free-living *T. calospora* respectively.

References

Cuomo CA, Bakkeren G, Khalil HB, et al (2017) Comparative Analysis Highlights Variable Genome Content of Wheat Rusts and Divergence of the Mating Loci. G3 (Bethesda) 7:361–376. https://doi.org/10.1534/g3.116.032797

Duplessis S, Cuomo CA, Lin Y-C, et al (2011) Obligate biotrophy features unraveled by the genomic analysis of rust fungi. Proc Natl Acad Sci USA 108:9166–9171. https://doi.org/10.1073/pnas.1019315108

Fochi V, Chitarra W, Kohler A, et al (2017a) Fungal and plant gene expression in the *Tulasnella calospora–Serapias vomeracea* symbiosis provides clues about nitrogen pathways in orchid mycorrhizas. New Phytologist 213:365–379. https://doi.org/10.1111/nph.14279

Fochi V, Falla N, Girlanda M, et al (2017b) Cell-specific expression of plant nutrient transporter genes in orchid mycorrhizae. Plant Science 263:39–45. https://doi.org/10.1016/j.plantsci.2017.06.015

Kämper J, Kahmann R, Bölker M, et al (2006) Insights from the genome of the biotrophic fungal plant pathogen Ustilago maydis. Nature 444:97–101. https://doi.org/10.1038/nature05248

Kohler A, Kuo A, Nagy LG, et al (2015) Convergent losses of decay mechanisms and rapid turnover of symbiosis genes in mycorrhizal mutualists. Nature Genetics 47:410–415. https://doi.org/10.1038/ng.3223

Krizsán K, Almási É, Merényi Z, et al (2019) Transcriptomic atlas of mushroom development reveals conserved genes behind complex multicellularity in fungi. PNAS 116:7409–7418. https://doi.org/10.1073/pnas.1817822116

Lanver D, Müller AN, Happel P, et al (2018) The Biotrophic Development of *Ustilago maydis* Studied by RNA-Seq Analysis. The Plant Cell 30:300–323. https://doi.org/10.1105/tpc.17.00764

Ohm RA, de Jong JF, Lugones LG, et al (2010) Genome sequence of the model mushroom *Schizophyllum commune*. Nat Biotechnol 28:957–963. https://doi.org/10.1038/nbt.1643

Ohm RA, Riley R, Salamov A, et al (2014) Genomics of wood-degrading fungi. Fungal Genet Biol 72:82–90. https://doi.org/10.1016/j.fgb.2014.05.001

Perotto S, Rodda M, Benetti A, et al (2014) Gene expression in mycorrhizal orchid protocorms suggests a friendly plant–fungus relationship. Planta 239:1337–1349. https://doi.org/10.1007/s00425-014-2062-x

Salcedo A, Rutter W, Wang S, et al (2017) Variation in the AvrSr35 gene determines Sr35 resistance against wheat stem rust race Ug99. Science 358:1604–1606. https://doi.org/10.1126/science.aao7294
